# Pragmatics as Social Inference About Intentional Action

**DOI:** 10.1162/opmi_a_00191

**Published:** 2025-02-08

**Authors:** Manuel Bohn, Michael C. Frank

**Affiliations:** Institute of Psychology in Education, Leuphana University Lüneburg, Lüneburg, Germany; Department of Comparative Cultural Psychology, Max Planck Institute for Evolutionary Anthropology, Leipzig, Germany; Department of Psychology, Stanford University, Stanford, CA, USA

**Keywords:** pragmatics, gesture, inference, communication

## Abstract

Pragmatic inferences are based on assumptions about how speakers communicate: speakers are taken to be cooperative and rational; they consider alternatives and make intentional choices to produce maximally informative utterances. In principle, this analysis applies to linguistic but also non-linguistic communicative actions, but this prediction is typically only tested in children and not in more systematic implicature contexts. We test key implications of this view across six online experiments with American English speaking adults (total *N* = 231). Experiments 1A and 1B showed that participants made pragmatic inferences based on different types of communicative actions, some being non-linguistic. In Experiment 2, pragmatic inferences were found to be conditional on the speaker’s epistemic states. Finally, Experiments 3A to 3C showed that pragmatic inferences were more likely to be made when the communicative action was produced intentionally. Taken together, these results strengthen the view that pragmatics includes social inference about cooperative communication over intentional actions, even non-linguistic actions.

## INTRODUCTION

Human communication is not reducible to the words and sentences being uttered: there is an inferential gap between what a speaker said and what they meant. Language users use pragmatic inferences—inferences about communicative intention in context—to close this gap and recover the intended meaning. As such, pragmatics is thought to have a central role in everyday language as well as in language learning (Bohn & Frank, 2019; Clark, 1996). Pragmatic inferences are computed in a social-cognitive reasoning process based on assumptions about how speakers communicate (Levinson, 2000; Sperber & Wilson, 1995). On a Gricean view, these assumptions entail that the speaker communicates in a rational and cooperative way (Grice, 1991). The speaker is assumed to consider multiple possible utterances from which they intentionally choose the one that is maximally informative to the listener.

According to one influential theoretical model—the Rational Speech Act framework—the pragmatic inference process can be described as follows (Degen, 2023; Frank & Goodman, 2012; Franke & Jäger, 2016; Goodman & Frank, 2016). The listener assumes that in order to communicate an intended meaning, the speaker considers multiple possible utterances. The speaker assesses how informative each utterance is—how likely it is to convey the intended meaning—given the context. Finally, the speaker intentionally chooses the most informative utterance. The listener works backwards through these steps and thereby recovers the speaker’s intended meaning. This view of pragmatic inference has led to a productive interdisciplinary research program covering a diverse range of topics such as reference resolution (Frank & Goodman, 2014), information integration (Bohn, Schmidt, et al., 2022; Bohn et al., 2021; Bohn, Tessler, et al., 2022), convention formation (Hawkins et al., 2023), semantic theories (van Tiel et al., 2021), and even non-human communication (Bohn, Liebal, et al., 2022; Franke et al., 2024).

Almost by definition, utterances are usually taken to be linguistic expressions (Sauerland, 2012). Yet, inspired by evolutionary and developmental thinking, the idea has been put forward that pragmatic inferences are more general and can be derived from all sorts of communicative actions, linguistic and non-linguistic^1^ (Heintz & Scott-Phillips, 2023; Moore, 2017; Sterelny, 2017; Tomasello, 2008). According to Sperber and Wilson (1995), any kind of action can be used communicatively as long as it is intentionally produced for this purpose (and recognized by the listener as produced that way) (see also Csibra & Gergely, 2009; Heintz & Scott-Phillips, 2023; Tomasello, 2008). As a consequence, pragmatic inferences should depend on intentionally produced non-linguistic aspects—movements, gestures, or eye gaze—that accompany utterances in just the same way as non-linguistic aspects; inferences should even be possible for entirely non-linguistic communicative actions. What matters from this perspective are the intentions and epistemic states that underlie the speaker’s production of the communicative action. Epistemic states in this context refer to the speaker’s perceptions, knowledge and beliefs that are the basis for their communicative action, such that e.g., a speaker will only communicate about an object they know about. Thus, when making inferences about the meaning of non-linguistic actions, speakers make simultaneous inferences about intentions and epistemic states.

This idea—that non-linguistic action is integrated into contextual reasoning—is commonplace in the developmental literature on children’s reasoning. For example, eye-gaze and gesture are widely believed to be critical information sources used for inferring word meanings in early childhood (e.g., Çetinçelik et al., 2021; Hollich et al., 2000; Kirk et al., 2022; Rowe et al., 2008). Yet these aspects of early contextual reasoning are not always integrated with work on the development of linguistic pragmatic inference, in particular the ability to make conversational implicatures (Barner et al., 2011; Katsos & Bishop, 2011; Noveck, 2001). Here we draw on the viewpoint that these abilities—early-developing contextual reasoning about reference and later developing linguistic pragmatics—are in fact continuous with one another (Bohn & Frank, 2019). This developmental work further supports the view articulated above, namely that both linguistic and non-linguistic actions support pragmatic inference.

Our goal in this paper is to experimentally test three key implications of this view, using adults’ pragmatic reasoning as our text case. These three implications are 1) that pragmatic inferences can be made over non-linguistic actions, 2) that these inferences depend on speakers’ epistemic states for both linguistic and non-linguistic actions, and 3) that they depend on the intentionality of the actions.

First, we test if pragmatic inferences can be derived from non-linguistic acts of communication alone. Non-linguistic cues such as gaze and pointing are known to complement language processing (Jachmann et al., 2023; Knoeferle & Kreysa, 2012; Staudte et al., 2014). Barr and Kelly (1997) showed that non-linguistic actions like gaze and pointing leads listeners to infer that an utterance is an indirect request instead of a simple assertion. Tieu and colleagues (2019) showed that adults make pragmatic inferences when parts of utterances are substituted by iconic gestures or animations. Kampa and Papafragou (2023; see also Kampa et al., 2024; Sullivan et al., 2022) showed that adults and children make pragmatic inferences based on drawings. In their study—which required a comparable pragmatic inference to the studies reported below—the sender was said to paint a picture of which of two boxes they saw. Both boxes contained the same two objects (e.g., pumpkin and a penguin) side by side. In the critical test trials, visual access to one object (the pumpkin) was blocked for the sender. When the sender drew a picture of the penguin, participants inferred that they saw the box with only the penguin visible because the pumpkin was only visible in the box with both objects visible and thus drawing it would have been the more informative way of indicating this box. We build on this work by studying the same basic inference and advance it by testing whether eye gaze or pointing gestures alone are sufficient to derive pragmatic inferences.

Second, we ask if listeners base their inferences on the speaker’s epistemic states. This question is central because what the speaker knows determines what alternative communicative actions they could have used. In other words, intentionally choosing to produce an action instead of an alternative requires being aware of the alternative. This step replicates and extends earlier work documenting the use of epistemic states in pragmatic inference (Bergen & Grodner, 2012; Breheny et al., 2013; Kampa & Papafragou, 2020; Katsos et al., 2023; Spychalska et al., 2021; Wilson et al., 2023).

Third and finally, we assess the importance for pragmatic inference of whether the non-linguistic aspects of a communicative act were produced intentionally or not. That is, we ask if receivers focus on the actions of the sender alone or whether they also take into account the intentions that generate these actions. While linguistic utterances are almost by definition produced intentionally for communicative purposes, non-linguistic communicative actions such as gaze and gestures are not. This aspect has not been studied intensely thus far and constitutes the most novel contribution of our work.

**Figure 1.**
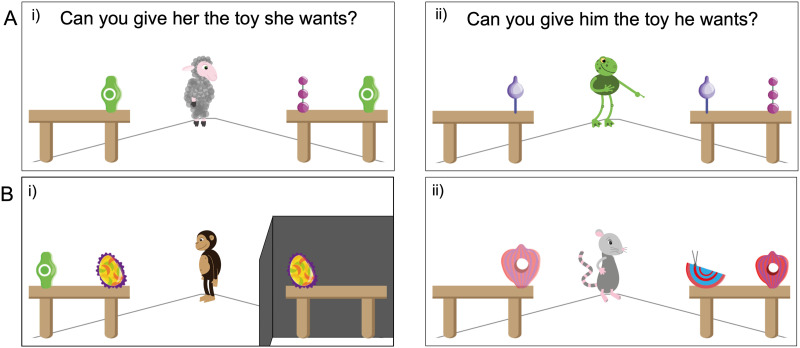
Screenshots from Experiments 1 and 2. Panel A shows the look (i) and point (ii) configuration of Experiment 1A and 1B. In the beginning of the experiment, the animal faced the participant and then turned to one of the tables and made a request. The point and gaze configuration were either paired with a verbal utterance or not to yield the four conditions (*look and label*, *look*, *point and label*, *point*). Panel B shows the two control conditions *barrier* (i) and *later* (ii) from Experiment 2. In the *barrier* condition, a barrier blocked the animal’s sight of the second table. In the *later* condition, one table was initially empty and only after the animal turned to the table with the two objects, the objects on the empty table appeared (object is depicted as slightly transparent to show that it is just appearing).

In our experimental setup, we focused on how listeners make pragmatic inferences to identify the referent of a novel word (Bohn, Tessler, et al., 2022; Frank & Goodman, 2014). As in previous work, a simple pictorial vignette showed a speaker requesting objects; in our case, the objects were lying on tables to either side of the speaker. On one table, there was an object of type A (henceforth: *alternative*) while on the other table was an object of type A and one of type B (henceforth: *target*, see Figure 1). The speaker always referred to the table with the two objects and—ambiguously—requested an object using a non-word (e.g., dax). Based on the definition of the pragmatic inference process outlined above, we expected listeners to reason as follows: the speaker had the option to refer to one of the two tables. The most informative way to refer to the alternative would have been to refer to the table with only the alternative on it. The speaker intentionally chose to refer to the other table instead which suggests that they want teh target. In the following six experiments we tested if this inference depends on the type of (non-)linguistic actions the speaker used (Experiments 1A and 1B), the speaker’s epistemic state (Experiment 2), and the intentional production of all aspects of the communicative action (Experiments 3A–3C).

## EXPERIMENT 1A

Our first experiment tested whether participants made similar pragmatic inferences from linguistic and non-linguistic actions, comparing 1) looking and labeling the object and 2) pointing at and labeling the object with 3) pointing alone and 4) looking alone. We predicted that participants would be equally likely to make pragmatic inferences based on non-linguistic actions (pointing or looking alone).

### Methods

The experiment was pre-registered at: https://osf.io/qcwr8. The code to run all experiments reported in this paper is available in the following public repository: https://github.com/manuelbohn/prag-int.

#### Participants.

Participants in all experiments were recruited via Amazon Mechanical Turk (MTurk) and required to have US IP addresses. We did not collect any additional demographic information such as age or gender. They received payment equivalent to an hourly wage of ∼$9. The sample size for all experiments (except Experiment 1B) was planned so as to have 120 observations (total number of trials) per cell. Participants were excluded when they failed to select known objects (a car and a ball) during training trials. The planned sample size for Experiment 1A was 60. Three participants did not provide valid data so that the final sample size for Experiment 1 was 57. Data was collected in March 2018.

#### Material.

All experiments were framed as word learning games in which animal characters would make requests and participants would have to select the corresponding objects. They were implemented as a website using HTML and JavaScript to which participants were directed via MTurk. Animals were cartoon figures that could be animated. Objects were novel objects drawn for the purpose of the study. For each animal, we recorded a set of utterances (one native English speaker per animal). The scene for all experiments involved the animal standing between two tables, one with two novel objects (A and B), and the other with one novel object (A) (see Figure 1A). Each trial involved a different animal and different objects. Participants responded by clicking on objects on the screen.

#### Procedure.

All experiments started with an introduction to the animals and two training trials in which familiar objects were requested (car and ball). Next, the animal turned to the table with the two objects (alternative and target). At this point, participants had to select which object they thought the animal had requested. The four conditions differed in how animals made the request. In the *look and label* condition—the reference condition—the animal turned towards the table with the two objects and said “Oh cool, there is a [non-word] on the table, how neat, can you give me the [non-word]?”. In the *point and label* condition, in addition to turning, the animal pointed towards the table while making the same request. In the *point* condition, the animal turned and pointed but did not utter the request. In the *look* condition, the animal only turned towards the table, without pointing or uttering. In all conditions, “Can you give [he/she] the toy she wants” was written above the animal as soon as they turned.

In all conditions, participants could infer that the animal requested the target via the counter-factual inferences that, if the animal had wanted to request the alternative, they would have pointed to the table with only the alternative (this being the most informative way to refer to the alternative). Thus, we coded as “correct” when participants selected the target. Participants completed two trials per condition and thus eight trials in total. They received no feedback about their choice. The order of conditions was randomized and the table on which the two objects were located was counterbalanced.

#### Analysis.

All analyses were run in R (R Core Team, 2024). To compare performance against a level expected by chance (50%), we used one-sample Bayesian *t*-tests via the function ttestBF from the BayesFactor package (Morey & Rouder, 2024). To compare conditions, we used Bayesian generalized linear mixed models fit via the function brm from the brms package (Bürkner, 2017). Regression models for all experiments included fixed effects for condition and random effects for participant and item with random slopes for condition within each of them (brms notation: correct ∼ condition + (condition ∣ participant) + (condition ∣ item)). All analysis used default priors implemented in the respective packages.

### Results

**Figure 2.**
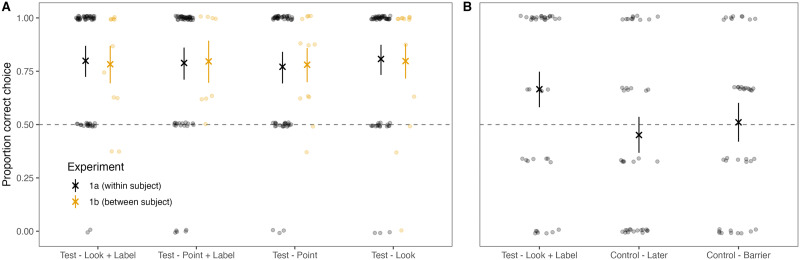
Results for Experiment 1A and 1B (A) and Experiment 2 (B). Transparent dots show aggregated data from individual participants (slightly jittered to avoid overplotting), crosses represent condition means, error bars are 95% CIs. Dashed line indicates performance expected by chance. Color in A shows the two experiments.

Participants performed above chance in all four conditions, that is, all Bayes Factors indicated very strong evidence in favor of the hypothesis that performance was different from 50% correct (Lee & Wagenmakers, 2014, see Table 1 and Figure 2). Performance in none of the conditions was reliably different (i.e., all 95% CrI included zero) from the *look and label* condition (reference condition); *point and label*: *β* = 0.29, 95% CrI (−0.75; 1.72); *point*: *β* = 0.10, 95% CrI (−0.92; 1.53); *look*: *β* = 0.26, 95% CrI (−0.64; 1.42). Bayes Factors for the direct comparisons to the *look and label* condition were all smaller than 1, suggesting moderate evidence in favor of the null hypothesis of no difference (*point and label*: 0.15, *point*: 0.17, *look*: 0.15; note that this analysis was not pre-registered).

**Table 1.** Comparison against chance level

Experiment	Condition	Mean	*SD*	BF
Experiment 1A	Test - Look	0.81	0.30	7.5e+07
	Test - Look + Label	0.80	0.28	1.3e+08
	Test - Point	0.77	0.30	1.9e+06
	Test - Point + Label	0.79	0.33	1.1e+06
Experiment 1B	Test - Look	0.80	0.32	8.52
	Test - Look + Label	0.78	0.25	13.56
	Test - Point	0.78	0.22	38.97
	Test - Point + Label	0.80	0.22	8.94
Experiment 2	Control - Barrier	0.51	0.37	0.17
	Control - Later	0.45	0.42	0.22
	Test - Look + Label	0.67	0.40	3.52
Experiment 3A	Control - Person	0.64	0.38	1.24
	Test - Look + Label	0.75	0.33	93.07
Experiment 3B	Control - Bell	0.59	0.34	0.5
	Test - Look + Label	0.74	0.29	303.12
Experiment 3C	Control - Tornado	0.53	0.32	0.23
	Test - Look + Label	0.80	0.28	7.5e+03

*Note*. BF = Bayes factor in favor of the hypothesis that performance is different from chance (50% correct).

### Discussion

The results of Experiment 1A showed that the way in which the speaker made a request, be it linguistic or non-linguistic or including pointing or not, did not affect participants’ inferences. In all conditions, performance was clearly above chance. This suggests that informativeness inferences are general inferences about (rational) intentional action and not bound to a particular class of communicative actions. For one thing, these findings corroborate previous work, showing that adults make pragmatic inferences based on gestures and drawings (Kampa & Papafragou, 2023; Tieu et al., 2019). For another, they also extend this previous work by showing that social cues like gaze and pointing—actions that are more frequent in everyday communication compared to drawings or iconic gestures—may serve the same function. However, an objection to this result could be that the similarities between conditions was a carry-over effect of our within-subjects design. That is, the inference might have carried over from e.g., the *look and label* conditions to conditions involving no labels. To rule out this objection, we conducted Experiment 1B.

## EXPERIMENT 1B

Experiment 1B was a between-subject replication of Experiment 1A. It was pre-registered at: https://osf.io/3vpqb.

### Methods

#### Participants.

The planned sample size for Experiment 1B was 60. Sixteen participants did not provide valid data so that the final sample size for Experiment 1B was 44. Data was collected in March 2018.

#### Material.

The material was the same as in Experiment 1A.

#### Procedure.

The procedure was identical to Experiment 1A, except that participants were randomly assigned to one of the four conditions.

#### Analysis.

The only difference in the analysis was that the regression model did not include random slopes for condition within subjects because subjects only completed one condition.

### Results

The results mirrored those of Experiment 1A. Performance was above chance (see Table 1 and Figure 2) and none of the conditions was reliably different from the *look and label* condition; *point and label*: *β* = 0.28, 95% CrI (−2.59; 3.04); *point*: *β* = −0.15, 95% CrI (−2.72; 2.40); *look*: *β* = 0.53, 95% CrI (−2.09; 3.18). Bayes Factors for the direct comparisons to the *look and label* condition were all smaller than 1 and suggested anecdotal evidence in favor of the null hypothesis of no difference (*point and label*: 0.41, *point*: 0.38, *look*: 0.38; note that this analysis was not pre-registered).

### Discussion

Experiment 1B suggests that the results of Experiment 1A do not reflect simple carry-over effects and supports the conclusion that informativeness inferences are computed independent of particular communicative actions.

## EXPERIMENT 2

In Experiment 2, we tested whether the pragmatic inference of Experiment 1 involves reasoning about the speaker’s epistemic states. The counter-factual inference involves that the speaker is aware of the alternative on both tables: roughly, if the speaker had wanted the alternative, they would have looked at the table with only the alternative on it (which they were aware of). Thus, if this would not be the case, the inference should go away. We tested this hypothesis by contrasting the *look and label* condition from Experiment 1A with two control conditions, one in which a barrier blocked the speaker’s view and one in which an object appeared only after the speaker made their request. Experiment 2 was pre-registered at https://osf.io/c2gjf.

### Methods

#### Participants.

The planned and achieved sample size for Experiment 2 was 40. Data was collected in May 2018.

#### Material.

The material was the same as in Experiment 1A.

#### Procedure.

The procedure for the *look and label* condition was identical to Experiment 1. In the *later* condition, the table containing only the alternative was empty when the animal turned and made their request. After the request was made but before participants could make a choice, the alternative appeared on the previously empty table. Thus, when selecting, the scene was identical to the *look and label* condition—but the speaker could not have taken into account the presence of the alternative on the other table when making the request. In the *barrier* condition, a black barrier was blocking the agent’s (but not the participants) view to the table with only the alternative (see Figure 1B). Participants completed three trials per condition and thus nine trials in total. They received no feedback about their choice. The order of trials was randomized and the table on which the two objects were located was counterbalanced.

#### Analysis.

The analysis was identical to Experiment 1A. The reference category for condition in the regression model was the *look and label* condition.

### Results

The Bayes factors suggest that performance was different from chance in the *look and label* condition but not in the two control conditions. (see Table 1 and Figure 2). Note that performance in the *look and label* condition was somewhat lower compared to Experiment 1A and 1B. We suspect this to be random fluctuation because the procedure and stimuli were identical to all other experiments. Importantly, however, the target was select less frequently in the two control conditions compared to the *look and label* condition; *later*: *β* = −2, 95% CrI (−3.78; −0.49); *barrier*: *β* = −1.39, 95% CrI (−2.66; −0.30).

### Discussion

Experiment 2 suggests that participants do indeed take the speaker’s epistemic state into account when computing informativeness inferences. In situations when the speaker was unaware of the alternative on the second table (and could thus not have referred to the alternative in a more informative way), participants did not make the inference that the speaker was referring to the target. As such, this result supports earlier work showing that listeners consider speaker’s epistemic states when making pragmatic inferences (Bergen & Grodner, 2012; Breheny et al., 2013; Kampa & Papafragou, 2020; Katsos et al., 2023; Spychalska et al., 2021; Wilson et al., 2023). As noted above, a second critical component of the inference is that the communicative action is produced intentionally. In Experiment 3, we varied whether or not the communicative action was an intentional action.

## EXPERIMENT 3A

In the previous experiment, before producing the verbal utterance, the speaker intentionally turned, gestured and gazed towards one of the tables. As outlined above, we see these non-linguistic actions as an essential part of the communicative action that play a crucial role in deriving the pragmatic inference. In the following three experiments, we systematically tested this idea by manipulating whether or not the speaker produced the movements and gestures preceding the verbal utterance intentionally and communicatively. In Experiment 3A, the agent’s turning was not part of an intentional communicative action: the agent turned towards the table with the two objects because a second agent appeared there. Experiment 3A was pre-registered together with 3b an 3c at: https://osf.io/nr3da.

### Methods

#### Participants.

The planned and achieved sample size for Experiment 3A was 30. Data was collected in May 2018.

#### Material.

**Figure 3.**
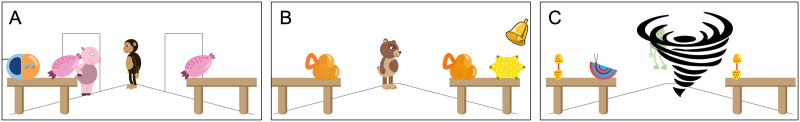
Screenshots from the three control conditions from Experiments 3A to 3C. In the person condition (A), the animal faced the participant, then another person (animal) appeared through the schematic door next to the table with the two objects saying “hi” upon which the speaker animal turned towards the person and the table with the two objects. Next, the object on the other table disappeared. Then the animal made the usual request. In the bell condition (B) instead of a person, a bell appeared and rang next to the table with the two objects. The following events were the same as in the person condition. In the tornado condition (C), the animal was lifted up into the air by a tornado, whirled around and landed facing the table with the two objects. The following events were the same as in the previous conditions.

The material was the same as in Experiment 1A except that a schematic door was added to the left and right of the speaker (see Figure 3).

#### Procedure.

The procedure for the *look and label* condition was identical to previous experiments except that after turning to the table with the two objects, the object on the other table disappeared. Only after that did the animal make the request. In the *person* condition, a second animal appeared next to the table with the two objects, saying “hi”. Next, the speaker animal turned towards this second animal (as if reacting to the “hi”). Then, the object on the table containing only the alternative disappeared and so did the second animal. The object was removed so that the speaker animal could not have turned back to the table with the single object in case they wanted to request the alternative. Then the speaker animal made the request. Thus, in both conditions, the test situation was identical (the speaker animal facing the table with the two objects while the other table was empty) but differed in whether or not the turning of the animal was apparently part of the communicative action or had a different potential explanation. Participants completed four trials per condition and thus eight trials in total. They received no feedback about their choice. The order of conditions was randomized and the table on which the two objects were located was counterbalanced.

#### Analysis.

The analysis was identical to Experiments 1A and 2. The reference category for condition in the regression model was the *look and label* condition.

### Results

**Figure 4.**
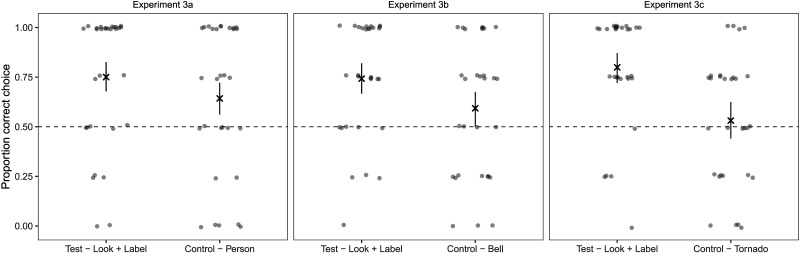
Results for Experiment 3A, 3B and 3C. Transparent dots show aggregated data from individual participants (slightly jittered to avoid overplotting), crosses represent condition means, error bars are 95% CIs. Dashed line indicates performance expected by chance.

Performance was clearly above chance in the *look and label* condition. There was considerably less evidence for performance being above chance in the *person* condition (see Table 1 and Figure 4). However, performance in the *person* condition was not reliably different from the *look and label* condition: *β* = −0.86, 95% CrI (−2.74; 0.86).

### Discussion

The results of Experiment 3A were inconclusive. Performance was numerically better in the *look and label* condition but it was not reliably so compared to the control condition. One potential explanation for this result could be that participants assumed the second animal’s actions were not independent of the speaker animal’s request but were deliberate attempts to draw the speaker animal’s attention to the table with the two objects, perhaps because this would be the one the speaker animal had a preference for. Of course, alternative explanations are possible. Thus, in Experiment 3B, we removed the second animal.

## EXPERIMENT 3B

### Methods

#### Participants.

The planned and achieved sample size for Experiment 3B was 30. Data was collected in May 2018.

#### Material.

The materials were the same as in Experiments 1A and 2. The schematic door introduced in Experiment 3A was removed (see Figure 3C).

#### Procedure.

The procedure for the *look and label* condition was identical to Experiment 3A, that is after turning to the table with the two objects, the object on the other table disappeared. In the *bell* condition, a bell appeared on the side with the table with objects A and B, accompanied by a ringing sound, upon which the animal turned to that side. Next, the object on the table containing only the alternative disappeared and the animal made the request. Once again, the test situation was identical in both conditions but differed in whether or not the turning of the animal appeared to be part of the utterance or whether it had a plausible alternative explanation. Participants completed four trials per condition and thus eight trials in total. They received no feedback about their choice. The order of conditions was randomized and the table on which the two objects were located was counterbalanced.

#### Analysis.

The analysis was identical to previous experiments. The reference category for condition in the regression model was again the *look and label* condition.

### Results

Performance in the *look and label* condition was clearly above chance. In contrast, there was no evidence in favor of this hypothesis in the *bell* condition (see Table 1 and Figure 4). When directly comparing the conditions, we found that performance was numerically better in the *look and label* condition but the 95% CrI overlapped with zero: *β* = −0.98, 95% CrI (−2.06; 0.07).

### Discussion

Experiment 3B provides tentative support for the idea that participants take into account whether or not a communicative action was produced intentionally. Yet, the results were not very strong. One reason for a weaker differentiation between the two conditions could be that the turning was still perceived as an intentional action, which might have coincided with the appearance of the bell. In Experiment 3C, we made it unambiguously clear that the turning towards the table with the two objects was not an intentional action.

## EXPERIMENT 3C

### Methods

#### Participants.

The planned and achieved sample size for Experiment 3C was 30. Data was collected in May 2018.

#### Material.

The material was the same as in Experiment 3B (see Figure 3C).

#### Procedure.

The procedure for the *look and label* condition was slightly modified: the animal first turned to the table with the two objects, then to the table with one object and then back again to the table with the two objects. Then the animal made the usual request. This turning was added to mimic the motion and timing of the *tornado* condition. Here, a tornado appeared and whirled the animal around multiple times and up into the air. Next the tornado disappeared, the animal landed on the ground facing the table with the two objects. When the animal landed, the object on the table containing only the alternative disappeared. Then the animal made the request. Once again, the test situation was identical in both conditions. Participants completed four trials per condition and thus eight trials in total. They received no feedback about their choice. The order of conditions was randomized and the table on which the two objects were located was counterbalanced.

#### Analysis.

The analysis was identical to previous experiments. The reference category for condition in the regression model was again the *look and label* condition.

### Results

Performance in the experiment was clearly above chance in the *look and label* condition but not the *tornado* condition (see Table 1 and Figure 4). When directly comparing the conditions, performance was reliably better in the *look and label* condition: *β* = −1.79, 95% CrI (−2.96; −0.82).

### Discussion

In Experiments 3A and 3B, the speaker intentionally turned towards the table with the two objects, albeit for reasons other than wanting to communicate about the objects. In Experiment 3C, when it was unambiguously clear that the movements and gestures preceding the verbal utterance were not produced intentionally, participants did not make the informativeness inference. Together with Experiments 3A and 3B, this result supports the interpretation that participants base their pragmatic inferences based on an assessment of the speaker’s intentions when producing the communicative actions and even suggests that participants are sensitive to the degree of intentionality involved in the production of communicative actions.

## GENERAL DISCUSSION

Across six experiments, we tested the implications of theories that construe pragmatic inference as a social-cognitive reasoning process about intentional communicative action. We used pragmatic word learning as a case study to test three predictions. First, we found that listeners make very similar pragmatic inferences based on linguistic and non-linguistic actions. This result supports the view that listeners make pragmatic inferences about many forms of communicative action, not just language. Second, consistent with prior work, we found pragmatic inferences to be conditional on what the speaker knows when producing a communicative action and thus which alternative actions they could have produced. Finally, we showed the importance of non-linguistic aspects of communicative actions: when the movements and gestures preceding the verbal utterance were not part of an intentional communicative act, the pragmatic inference was lost.

Previous work with adults has shown that such cues complement language processing (Jachmann et al., 2023; Knoeferle & Kreysa, 2012; Smith et al., 2017; Staudte et al., 2014) or enable the derivation of pragmatic inferences (Barr & Kelly, 1997; Enfield, 2009; see also Engle, 1998). Experiments 1A and 1B extend this earlier work by showing that non-linguistic cues such as gaze and pointing gestures can be sufficient to elicit informativeness inferences. Experiment 2 conceptually replicates earlier work showing that listeners consider speakers’ epistemic states when making pragmatic inferences (Bergen & Grodner, 2012; Breheny et al., 2013; Kampa & Papafragou, 2020; Katsos et al., 2023; Spychalska et al., 2021; Wilson et al., 2023). Furthermore, the study presented here introduces two easy-to-implement paradigms to this field of research.

Finally, Experiments 3A to 3C explored a previously understudied aspect of pragmatic inferences: the role of intentionality in the production of a communicative action. One reason this aspect has been largely overlooked is the widespread focus on spoken language, where linguistic utterances are (almost) always produced intentionally, that is, with an intention to communicate. Non-linguistic cues, on the other hand, are more likely to be produced without such intentions, as mere by-products of other actions. The results reported here show that listeners take into account whether non-linguistic communicative actions were produced intentionally. Only when they were considered to be produced intentionally did they influence the interpretation of the entire communicative action. These results thereby contribute to connecting work on pragmatic inferences to the literature emphasizing the role of intentionality in the evolution of uniquely human forms of communication (Dennett, 1983; Tomasello, 2008; Townsend et al., 2017). By demonstrating that pragmatic inferences are contingent upon the intentional production of non-linguistic actions, we underscore the significance of communicative intentions in shaping the interpretation of communicative actions. This finding aligns with theoretical frameworks that emphasize the cooperative nature of communication, wherein speakers actively manipulate signals to convey specific meanings to listeners (Grice, 1991).

Our analysis of the reasoning process underlying pragmatic inferences has been inspired by computational cognitive models within the RSA framework (Degen, 2023; Frank & Goodman, 2012; Franke & Jäger, 2016; Scontras et al., 2021). Recent modeling studies have shown how the pragmatic inference processes described here interact with social-contextual information (i.e., common ground) during language learning and comprehension (Bohn et al., 2021; Bohn, Tessler, et al., 2022). Together with the findings reported here, these results highlight the value of computational models for explicating the cognitive processes that underlie communicative interactions.

Our study has important limitations. First, our case study was a single pragmatic inference about word meaning. This phenomenon has been studied in prior work and presented a convenient case study, but future work should ideally devise ways to test the same manipulations in the context of other types of pragmatic inference. Additionally, we tested a convenience sample of English-speaking adults with a US IP address. Our parameter estimates do not represent measures of any particular population, and whether the results would generalize to other languages or cultural settings is an open question. Since we make our materials openly available, adaptation to other languages would be relatively easy [and indeed German versions of these tasks yield interpretable data with children; Bohn et al. (2023)]. Furthermore, it would be interesting to conduct comparable experiments with children to better understand the developmental trajectories giving rise to the pattern reported for adults.

Taken together, this study highlights how experimental, computational, and developmental approaches can jointly elucidate the mechanisms underlying pragmatic inferences and, more broadly, human communication.

## FUNDING INFORMATION

Manuel Bohn was funded by a Jacobs Foundation Research Fellowship (Grant No. 2022-1484-00) and a Lower Saxony Impulse Professorship through the zukunft.niedersachsen program.

## AUTHOR CONTRIBUTIONS

Manuel Bohn: Conceptualization, Formal analysis, Writing – original draft, Writing – review & editing. Michael C. Frank: Conceptualization, Writing – original draft, Writing – review & editing.

## Note

^1^ Here we use the term “non-linguistic” to denote actions such as pointing, eye-gaze, and other gestures. These are sometimes referred to as “non-verbal” communicative gestures but this label is confusing in the context of signed languages.
